# Non-invasive near-infrared spectroscopy assessment of the spinal neurovascular response in a patient with transverse myelitis: a case report

**DOI:** 10.1186/s12883-022-02881-1

**Published:** 2022-10-24

**Authors:** Juan Esteban Oyarzún, Raúl Caulier-Cisterna, Juan Pablo González-Appelgren, Leticia Gonzalez, Oscar Trujillo, Antonio Eblen-Zajjur, Sergio Uribe

**Affiliations:** 1 Centro de Imágenes Biomédicas, Pontificia Universidad Católica de Chile, Santiago, Chile; 2 Rielo Institute for Integral Development, New York, USA; 3 Neurology Department, School of Medicine, Pontificia Universidad Católica de Chile, Santiago, Chile; 4 Laboratorio de Neurociencia Traslacional, Facultad de Medicina, Universidad Diego Portales Santiago, Santiago, Chile; 5 Radiology Department, School of Medicine, Pontificia Universidad Católica de Chile, Santiago, Chile

**Keywords:** Near infrared spectroscopy (NIRS), Spinal neurovascular response, Non-invasive method, Transverse myelitis, Spinal neuronal activity, Neuron-vascular coupling

## Abstract

**Background:**

Transverse myelitis (TM) is characterized by acute development of motor, sensory and autonomic dysfunctions due to horizontally diffused inflammation in one or more segments of the spinal cord in the absence of a compressive lesion. The not well-known inflammation process induces demyelination resulting in neurological dysfunction.

**Case presentation:**

In this case report we used a functional Near-Infrared Spectroscopy (fNIRS) technique to evaluate changes in the peri-spinal vascular response induced by a peripheral median nerve electrical stimulation in a patient with chronic transverse myelitis (TM). fNIRS showed drastically reduced signal amplitude in the peri-spinal vascular response, compared to that obtained from a healthy control group throughout most of the C7-T1 and T10-L2 spinal cord segments.

**Conclusion:**

The potential use of this relatively non-invasive fNIRS technology support the potential clinical application of this method for functional test of the spinal cord through the assessment of the spinal neurovascular response.

## Background

Transverse myelitis (TM) is characterized by acute motor, sensory and autonomic dysfunctions due to the horizontally diffused inflammation in one or more segments of the spinal cord in the absence of a compressive lesion [1]. The inflammation process that induces the demyelination that results in neurological dysfunction is not well-known [2]. In severe cases, 50% of TM patients lose movement of the legs, most have bladder dysfunction, and some exhibit paresthesia and/or dysesthesia of different kinds [3]. Longitudinal studies of TM reveal that one third of patients recover from these symptoms and signs, one third develop a moderate degree of permanent disability, and one third show permanent and severe disabilities [4]. This last group is characterized by a rapid initial progression of symptoms [5]. Diagnosis requires demonstration of spinal cord inflammation evidenced by an abnormal increase in gadolinium-MRI spinal signals combined with an elevated IgG index in the cerebrospinal fluid (CSF) [2].

Currently, MRI or computed tomography assessment of the spinal cord is mainly structural, however, they provide little information on spinal function [6]. At the present, the main functional spinal cord tests are clinical examination and evoked potentials (EP) with spinal emphasis, the latter based on the neuronal response associated with peripheral stimulation [7]. However, the low signal-to-noise ratio of EP requires prolonged, repetitive stimulation, and long-term averaging techniques to obtain clinically relevant results. Furthermore, EP maintains high levels of false negative or positive errors in addition to its annoying effects on the patients [8].

fNIRS is commonly used for the experimental evaluation of brain activity, as it can estimate changes in the concentration of oxyhemoglobin (HbO_2_) and deoxyhemoglobin (Hb), which is directly correlated with neuronal activity [9]. Animal studies support the use of fNIRS as a spinal blood perfusion monitor, both in direct injuries and surgical interventions at the spinal cord [10] but also for the detection of spinal ischemia during spinal cord-risk surgeries [11]. These reports support the need to develop a non-invasive spinal fNIRS technique as a potential tool to evaluate the hemodynamics of the spinal cord [12] and its changes induced by injuries. Recently, a novel non-invasive application of a fNIRS to assess the neurovascular response (NVR) of the human spinal cord has been proposed [13, 14]. In this report, we describe for the first time, the spinal cord NVR alterations in a TM patient applying a non-invasive fNIRS technique [13, 14].

## Case presentation

The subject is a 43-year-old woman, diagnosed with TM 11 years ago. The first symptoms were right upper pain and legs weakness, more intense while standing, and overall tiredness. At week 3, the patient showed loss of motor reflexes, ataxia and photosensitivity, reaching the maximum intensity by the 2nd month when a spinal cord lesion was detected by a non-contrasting MRI. At month 3, TM diagnosis was confirmed by CSF immunology, neurological symptoms and gadolinium-MRI showing a multisegmented demyelinating lesion. After clinical and laboratory general tests, comorbidities were diagnosed, i.e., von Willebrand disease, Ehlers-Danlos syndrome, autonomic dysfunction, sulcus vocalis, and a thyroid nodule. Currently, 11 years after the initial diagnosis of TM, the patient reports diffuse mid to severe multiarea body pain (VAS = 6 to 9), and paresis 2/5 (MRC) on the body right side. The patient requires a wheelchair for mobility and long-term care. Daily medication includes oral morphine and pregabalin, and topical lidocaine (Table 1).

**Table 1:** Table of clinical and anthropometric measures of the patient compared to Control Group (CG). F: Female; M: Male; BMI: Body Mass Index; NCS MN: Nervous Conduction Speed Median Nerve

Clinical data	Patient	Control group (n = 37)
Fnnirs evaluation date	01/31/2020	–
Gener (f, prop)	F	15 (0.36)
Age (years)	43	35.6 ± 17.3
Height (cm)	166	169.1 ± 9.86
Weight (kg)	85	72.5 ± 14.8
BMI (Kg.m^-2^)	30.8	25.2 ± 4.2
Body fat (%)	26.13	19.4 ± 5.4
Primary pathologies	Transverse myelitis	No disease
Secondary pathologies	Von willebrand’s disease, ehlers-danlos syndrome, autonomic dysfunction, sulcus vocalis and thyroid nodule	No disease
Current medication (doses)	Morphine (36 mg/day), pregabalin (375 mg/day), omeprazole (40 mg/day), amitriptyline (75 mg/day), tranexamic acid (500 mg/day), fludrocortisone (0.2 mg/day), roll on lidocaine	No medications
Median ncv (m.s^−1^)	54.6	49.8 ± 3.5
Right n_11_ latency	No tested	–
Left n_11_ latency	10.83	11.79 ± 1.1

**Fig. 1:**
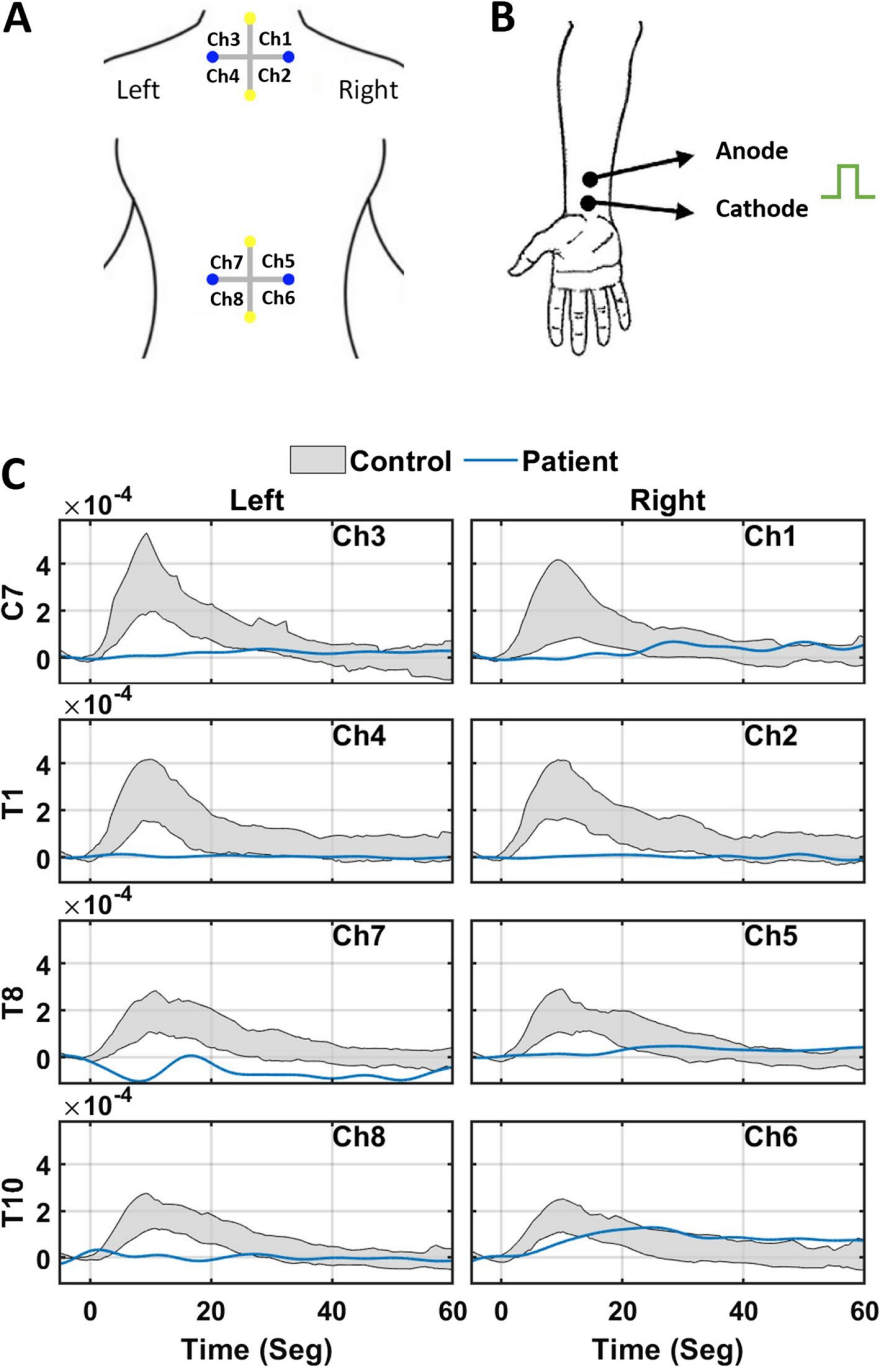
**A** Positioning scheme of superficial optopods. Yellow dots are laser light emitters and blue dots are optical receivers. Ch: channel. **B** Configuration of the superficial electrical stimulation at left median nerve. **C** NVR register by fNIRS in the eight channels scheme (**A**). Each channel measures the amplitude of the oxyhemoglobin signal versus the recording time, where zero corresponds to the peripheral stimulation caused in the left wrist (**B**). The record line of each patient corresponds to the average of the 3 pulses of the protocol. While the gray area shows the 20–80 percentile of the average response of each individual in the CG. Ch: channel

NVR was triggered by non-invasive electrical stimulation of the left median nerve, using a bipolar transcutaneous electrical stimulator (square, 10 mA, 5 ms, cathode proximal and 3 cm from anode) applied to the skin using electroconductive gel, over the nerve at the wrist medial midline. An array of 4 light sources and 4 photodetectors totaling 8 NIRS channels with a source-detector optodes distance of 4.5 cm were used to record the concentration changes of oxyhemoglobin (Δ [HbO_2_]) and deoxyhemoglobin (Δ [Hb])(Fig. 1A). The peri-spinal vascular response was measured from the upper (C_7_ and T_1_) and lower (T_10_ and L_2_) spinal cord, which were selected due to the cervical and lumbar sensory enlargement of the spinal cord (Fig. 1A) [13, 14].

A commercially available fNIRS device (Oxymon, Artinis^TM^, The Netherlands) designed for brain fNIRS and based on a continuous wave technology was modified to record spinal NVRs. The system uses time multiplexed method to combine sources and detectors without interference, with dual wavelengths of 760 nm and 850 nm i.e., under and above hemoglobin spectral isosbestic point within < 1 pW sensitivity.

In a quiet experimental room, i.e., < 30 db sound level, 5 × 4 square meter space and dimmed lighting, the patient was informed about the test, then instructed to lie down in prone position on a therapeutic/massage bed with the face inside the window of the bed. The wrist and back skin were cleaned with alcohol skin swabs. The optode holders were fixed tightly on to the skin at cervical and thoracic vertebral level with clinical skin adhesives using the vertebral spinous processes as anatomical landmarks.

The recording protocol consisted of applying 3 electrical stimuli, each of 10 mA every 4 minutes (Fig. 1B). The rise time, amplitude and duration of the NVR (raw optical density values) induced by each stimulus were measured and off-line processed i.e., filtering, displaying and saved for non-parametrical statics as described in detail elsewhere [11]. Patient recordings were made at least 3 hours after the last routine medication.

Median Nerve Conduction Velocity (NCV) was relatively normal (Table 1), left spinal N_11_ wave latency was 100% delayed, whereas the right one was not tested due patient condition. Median values of patient NVR data were compared to those obtained from a healthy control group (*N* = 37) (Table 1). The peri-spinal NVR of the patient was absent at C_7_ and T_1_ on both sides and for both sides of T_8_ as well as the left side of T_10_. An abnormally small delay of NVR was detected for the T_10_ right side (Fig. 1C). These findings agree with the notion of a diffuse and severe functional impairment of the neuron-vascular coupling process of the spinal cord correlated to the spinal inflammatory damage. The fNIRS record is consistent with almost complete paresis of the left and right sides of the body.

## Discussion and conclusions

The results reported here strongly suggest the ability of the non-invasive fNIRS assessment of the peri-spinal NVR to detect the functional alteration in the TM patient. The regional blood flow in a neuronal population is tight- and directly associated to the neuronal metabolic rate and activity, process known as neuro-vascular coupling [9, 11, 14] which take place at spinal dorsal horn [11] due to the input of peripheral sensory signals. Any neuronal and/or neuronal network disfunction induces alterations in the NVR [9, 11, 14], which shows the potential diagnostic application of this method for functional assessment of the spinal cord. The main source of the recorded NR is the peri-spinal vascular response without contribution of muscle activity as reported previously [13].

Despite that the BMI for the patient was higher than that for healthy controls, Valenzuela et al., 2021 (11) did not find statistical association between subjects’s BMI and spinal NVR parameters such as amplitude, rise time or duration, thus, contribution of the BMI to the spinal NVR in this case should be marginal [11].

These results strongly support the application of the new fNIRS to assess the spinal NRV in TM and its potential use on functional assessment of the human spinal cord, and opens a wide range of possibilities of examining other spinal inflammatory diseases, edema from different etiology, pharmacological effects, and ischemic lesions at the spinal cord in the same way that affects the brain [15].

## Data Availability

The raw data supporting the conclusions of this article will be made available by the authors, without undue reservation. To obtain the raw data contact the corresponding author (suribe@uc.cl).

## Abbreviations

TM: Transverse myelitis
fNIRS: functional Near-Infrared Spectroscopy
NIRS: Near Infrared Spectroscopy
CSF: Cerebrospinal fluid
MRI: Magnetic resonance imaging
HbO_2_: Oxyhemoglobin
Hb: Deoxyhemoglobin
NVR: Neurovascular response
